# Non-invasive prediction of preterm birth in women with cervical insufficiency or an asymptomatic short cervix (≤25 mm) by measurement of biomarkers in the cervicovaginal fluid

**DOI:** 10.1371/journal.pone.0180878

**Published:** 2017-07-10

**Authors:** Ha-Na Yoo, Kyo Hoon Park, Eun Young Jung, Yu Mi Kim, Song Yi Kook, Se Jeong Jeon

**Affiliations:** Department of Obstetrics and Gynecology, Seoul National University College of Medicine, Seoul National University Bundang Hospital, Seongnam, Korea; University of Illinois at Urbana-Champaign, UNITED STATES

## Abstract

**Objective:**

To determine whether various proteins in the cervicovaginal fluid (CVF) known to be involved in immune regulation, alone or in combination with clinical risk factors, can predict spontaneous preterm delivery (SPTD) in women with cervical insufficiency or a short cervix (≤25 mm).

**Methods:**

This retrospective cohort study included 62 asymptomatic women with cervical insufficiency (n = 27) or an asymptomatic short cervix (n = 35) at 18–27 weeks. CVF swab samples were taken for assays of vitamin D binding protein (VDBP), interleukin (IL)-8, matrix metalloproteinases (MMP)-9, tissue inhibitor of metalloproteinases (TIMP)-1, and Dickkopf-related protein 3 (DKK3) before cervical examination, and maternal blood was collected for the determination of the C-reactive protein (CRP) level. The primary outcome measurement was SPTD at <32 weeks of gestation. Logistic regression analysis and receiver operating characteristic curves were used for the statistical analyses.

**Results:**

The rate of SPTD at <32 weeks was 40.3% (25/62). The CVF levels of VDBP, TIMP-1, and DKK3, but not IL-8 and MMP-9, were significantly higher in the women who had SPTD at <32 weeks than in those who did not deliver spontaneously at <32 weeks. The women who had SPTD at <32 weeks had a significantly more advanced cervical dilatation at presentation and a higher level of serum CRP. Using the stepwise regression analysis, a prediction model was developed by combining various proteins in the CVF and clinical factors, resulting in the inclusion of cervical dilatation, CVF VDBP, and use of corticosteroids (area under curve, 0.909).

**Conclusions:**

In women with cervical insufficiency or a short cervix, VDBP, TIMP-1, and DKK3 in the CVF may be useful as non-invasive predictors of SPTD at <32 weeks. A combination of these markers and clinical factors appears to improve the predictability of SPTD compared with the markers alone.

## Introduction

Cervical insufficiency complicates 0.1–1% of all pregnancies and is thought to be the main cause of second trimester pregnancy loss or early preterm birth [1–3]. Despite the clinical relevance of cervical insufficiency, little is known regarding the pathophysiology involved and the preterm risk assessment tools, especially those involving non-invasive methods. Such information is relevant for the development of more-effective treatments targeting individual risks and for patient counseling.

Accumulated research data indicated that in flammatory and immune responses are involved in the mechanisms responsible for premature cervical ripening and dilatation, which are the predominant features in women with cervical insufficiency/short cervix, as the result of an extensive cervical remodeling [4, 5]. Importantly, several studies clearly demonstrated that important mediators implicated in the cervical remodeling process, including interleukin (IL)-6, IL-8, matrix metalloproteinases (MMP)-9, tissue inhibitor of metalloproteinases (TIMP)-1, and macrophage colony-stimulating factor (M-CSF), were important factors for predicting spontaneous delivery at term and preterm [6–11]. Moreover, recent studies demonstrated that the increased level of vitamin D binding protein (VDBP) in the cervicovaginal fluid (CVF) was associated with spontaneous labor both at term and preterm [12, 13]. However, to date, there is a paucity of information on the role of these proteins, particularly in the CVF compartment, in the prediction of preterm birth in women with cervical insufficiency/short cervix. On the other hand, although Dickkopf-related protein 3 (DKK3) is much known to function as a positive regulator of Wnt signaling and a tumor suppressor through inducing apoptosis [14, 15], none is known about its speculative effect in preterm birth.

It is generally considered that cervical insufficiency/sufficiency might be a continuum, using transvaginal sonographic examination of the cervix and a mid-trimester short cervix is associated with cervical insufficiency and subsequent preterm birth [16, 17]. The purpose of the study was to determine whether VDBP, IL-6, IL-8, MMP-9, TIMP-1, M-CSF, and DKK3 levels in the CVF, alone or in combination with clinical risk factors, can predict spontaneous preterm delivery (SPTD) in women with cervical insufficiency or a short cervix (≤25 mm). Additionally, we compared these protein concentrations in the CVF compartment between women with cervical insufficiency and a short cervix.

## Materials and methods

### Study design

This was a retrospective cohort study conducted at Seoul National University Bundang Hospital (Seongnamsi, Korea), a tertiary-care teaching hospital, from September 2009 to December 2015. The patients consecutively recruited in this study were singleton pregnant women with a diagnosis of cervical insufficiency (painless cervical dilatation) or a short cervix between 18+0 and 27+6 weeks of gestation. The inclusion criteria were as follows: 1) a live fetus, 2) intact amniotic membranes, and 3) a CVF sample available for analysis. The exclusion criteria were as follows: multiple pregnancy, prior cervical cerclage, major congenital anomalies, preterm labor, or preterm premature rupture of the membranes at diagnosis, vaginal bleeding, and clinical signs of chorioamnionitis. Cervical insufficiency was defined as a painless cervical dilation of ≥1 cm and exposed fetal membranes determined via visual evaluation using a sterile speculum, without contractions of the uterus. A short cervix was defined as a cervical length of ≤25 mm measured via transvaginal ultrasound. Gestational age was calculated based on the last menstrual period and first trimester or second trimester (≤20 weeks) ultrasound results when available. However, in most of the study subjects, gestational age was confirmed using ultrasonography in the first or second trimester. This study was approved by the Institutional Review Board of Seoul National University Bundang Hospital (project number B-1105/128-102). The patients provided written informed consent for the collection and use of the CVF samples for research purposes. The primary outcome measure was SPTD at <32 weeks of gestation; SPTD was defined as delivery after spontaneous onset of preterm labor, premature rupture of membranes, or development of clinical chorioamnionitis.

### CVF

At the time of admission, CVF samples were obtained from all participants. CVF sample collection, processing, and storage have been previously described in detail [18]. Briefly, under sterile speculum examination, CVF samples were collected from the posterior vaginal fornix using two sterile Dacron swabs (Puritan Medical, Guilford, ME, USA) placed for 15 seconds to absorb the cervicovaginal secretions. The two Dacron swabs were then placed in two cryotubes containing 1 mL of sample buffer each and stored at -70°C for further analysis. C-reactive protein (CRP) in maternal blood was usually measured within 2–3 hours of sampling.

#### Analysis of various proteins in the CVF

The CVF samples were assayed for various proteins (IL-6, IL-8, VDBP, MMP-9, TIMP-1, M-CSF, and DKK3) using an enzyme-linked immunosorbent assay human DuoSet Kit (R&D Systems, Minneapolis, MN, USA) according to the manufacturer’s instruction. The ranges of the IL-6, IL-8, VDBP, MMP-9, TIMP-1, M-CSF, and DKK3 standard curves were 7.8–600, 31.2–2000, 156–10000, 31.2–2000, 31.2–2000, 15.6–1000, and 31.2–2000 pg/mL, respectively. Prior to the measurement of these seven proteins, the CVF samples were diluted using the ratio 1:4 for DKK3 and M-CSF, 1:5 for IL-6 and IL-8, 1:100 for MMP-9 and TIMP-1, and 1:500 for VDBP. In the samples with protein concentrations lower than the lowest point on the standard curve, the lowest detected values were used for the analysis. All the samples were assayed in duplicate. The intra- and inter-assay coefficients of variation were <10% for all analyzed biomarkers.

### Management of cervical insufficiency or a short cervix and definitions of various factors

The detailed description of cervical length measurements was published elsewhere [19]. Management of patients with cervical insufficiency or a short cervix has been previously described in detail [20, 21]. In brief, rescue cerclage was offered to women who present with cervical insufficiency and the McDonald technique for performing cerclage was usually done under spinal anesthesia. For women with advanced cervical dilatation and bulging membranes, amnioreduction was performed to decrease intra-amniotic fluid pressure. All women with cervical insufficiency were administered prophylactic antibiotics. Tocolytic therapy (magnesium sulfate, ritodrine or atosiban) was administered at the discretion of the attending obstetrician when regular uterine contractions had developed after the cerclage procedure. Decisions regarding the treatment for a short cervix, such as progesterone supplementation, placement of a cervical cerclage, and antibiotic treatment, were left to the discretion of attending obstetrician. The enrolled women or attending obstetrician were not blinded to the sonographic cervical lengths. Although regimen for antibiotic prophylaxis in these patients varied according to study period and obstetricians, ampicillin and azithromycin (clarithromycin or erythromycin) were the main antibiotic used. Antenatal corticosteroids were administered between 24 and 34 weeks of gestation. Acute histologic chorioamnionitis was defined as the presence of acute inflammatory change in any tissue sample (amnion, chorion-decidua, umbilical cord, or chorionic plate) with the use of criteria previously published [22]. Clinical chorioamnionitis was defined as a body temperature > 37.8°C on 2 occasions at least 4 hours apart, and ≥ 2 of the following criteria: uterine tenderness; malodorous vaginal discharge; maternal leukocytosis (> 15,000/mm^3^); maternal tachycardia (> 100 beats/min); and fetal tachycardia (> 160 beats/min), following the recommendations of Gibbs et al. [23]

### Statistical methods

Statistical analyses were performed using the SPSS version 22.0 for Windows (IBM SPSS Statistics, Chicago, IL, USA). The Shapiro-Wilk test was used to assess whether data are normally distributed. Continuous data were analyzed using the Student’s t-test or Mann-Whitney U test, while categorical data were compared using the *χ*^2^-test or Fisher’s exact test, as appropriate. A multivariate logistic regression analysis was then performed using the forward stepwise technique to determine the best combination model for the prediction of SPTD. Linearity was checked by inspecting the scatterplot of the relationship between the dependent variable and the independent variable before performing the regression analysis. Variables with a *P*-value of <0.05 from the univariate analysis were entered into the logistic regression model, and a *P*-value <0.05 was required for the final inclusion in the model. Receiver operating characteristic (ROC) curves for the prediction of SPTD at <32 weeks were generated for each protein and used to identify the best cutoff values for each variable. The sampling-to-delivery interval was assessed using the Kaplan-Meier analysis and was compared between the groups using the log-rank test. Women who delivered preterm for maternal or fetal indications and those who were lost to follow-up were included in this analysis, with a censoring time equal to the sampling-to-delivery interval. The areas under the ROC curves (AUC) were computed for each protein and the best predictive model and compared using the method of DeLong et al. [24] The Spearman rank correlation test was used to measure the relationship between the continuous variables, which did not follow a normal distribution. All statistical analyses were performed using a two-sided test with a significance level of 0.05.

## Results

Of the 64 women who fulfilled the inclusion criteria, 2 women received a history-indicated cerclage, leaving 62 women in the final analysis, including 27 women with cervical insufficiency and 35 women with an asymptomatic short cervix. The median gestational age at the time of sampling was 22+5 weeks (range, 18+0 to 27+6 weeks). SPTD at <32 weeks of gestation occurred in 40.3% (25/62) of the patients.

IL-6, IL-8, VDBP, MMP-9, TIMP-1, M-CSF, and DKK3 were detected in the CVF in 48%, 98%, 84%, 94%, 82%, 43%, and 66% of the subjects, respectively. IL-6 and M-CSF were detected in lower quantities (undetectable in >50%) and were subsequently excluded from further analysis. The levels of IL-8, VDBP, MMP-9, TIMP-1, and DKK3 were significantly correlated with each other (all variables, r = 0.328–0.727, *P*<0.01), except for MMP-9 and DKK3. Cervical dilatation was also significantly correlated with VDBP, TIMP-1, and DKK3 (all variables, r = 0.366–0.618, *P*<0.005) but not the levels of IL-8 (r = -0.005, *P* = 0.971) or MMP-9 (r = -0.223, *P* = 0.081). However, there was no correlation between any of the measured protein levels in the CVF and gestational age at sampling.

The clinical and laboratory characteristics of the study population stratified according to SPTD at <32 weeks of gestation are presented in Table 1. The women who had SPTD at <32 weeks had a significantly more advanced cervical dilatation, higher proportion of cervical insufficiency, higher level of serum CRP, and higher rate of corticosteroids administration and were less likely to receive progesterone therapy than those who did not deliver spontaneously at <32 weeks. The median concentrations of VDBP, TIMP-1, and DKK3, but not IL-8 and MMP-9, were significantly higher in the women who had SPTD at <32 weeks than in those who did not deliver spontaneously at <32 weeks. However, no significant associations were found between SPTD at <32 weeks and maternal age, parity, gestational age at sampling, rescue cerclage placement, maternal white blood cell count, and use of tocolytics and antibiotics.

**Table 1:** Demographic, clinical, and laboratory characteristics of the study population.

Characteristics	Delivery <32weeks (n = 25)	Delivery ≥32weeks (n = 37)	*P*-value
Age (years)	31.7 ± 4.2	32.5 ± 4.2	0.526
Nulliparity	48% (12)	49% (18)	0.960
Gestational age at sampling (weeks)	22.3 ± 2.4	23.1 ± 2.5	0.248
Gestational age at delivery (weeks)	25.2 ± 3.7	37.4 ± 2.1	<0.001
Disease entity Cervical insufficiency Short cervical length	76.0% (19) 24.0% (6)	21.6% (8) 78.4% (29)	<0.001
Cerclage placement	48.0% (12)	59.5% (22)	0.374
Vaginal progesterone therapy	28.0% (7)	56.8% (21)	0.026
WBC count (thousand/mm³)	11.47 ± 3.73	10.85 ± 2.98	0.681
Serum CRP (mg/dL)	1.49 ± 1.76	0.37 ± 0.36	0.002
Cervical length by ultrasound (mm) in women with a short cervix	8.6 ± 5.4 (n = 6)	11.4 ± 6.4 (n = 29)	0.379
Cervical dilatation (cm) >2 cm ≤2 cm	3 (0–6)	0 (0–4)	<0.001
	60.0% (15)	5.4% (2)	<0.001
	40.0% (10)	94.6% (35)	
Cervicovaginal IL-8 (ng/mL)	11.44 ± 21.70	8.95 ± 15.19	0.113
Cervicovaginal VDBP (μg/mL)	1.86 ± 1.54	0.74 ± 0.81	0.002
Cervicovaginal TIMP-1 (ng/mL)	145.45 ± 190.13	42.31 ± 46.45	0.001
Cervicovaginal MMP-9 (ng/mL)	48.54 ± 36.88	67.88 ± 61.76	0.362
Cervicovaginal DKK3 (ng/mL)	1.28 ± 2.75	0.45 ± 0.55	<0.001
Percentage above LLOQ for cervicovaginal IL-6	76.0% (19)	29.7% (11)	<0.001
Percentage above LLOQ for cervicovaginal M-CSF	56.0% (14)	35.1% (13)	0.104
Use of tocolytics	52.0% (13)	35.1% (13)	0.187
Use of corticosteroids	56.0% (14)	29.7% (11)	0.039
Use of antibiotics	92.0% (23)	73.0% (27)	0.063
Clinical chorioamnionitis	24.0% (6)	0% (0)	0.003
Histologic chorioamnionitis^a^	75.0% (18/24)	22.2% (2/9)	0.013

Values are given as the means ± SDs, medians (ranges), or % (n). WBC, white blood cell; CRP, C-reactive protein; IL, interleukin; VDBP, vitamin D binding protein; TIMP, tissue inhibitor of metalloproteinases; MMP, matrix metalloproteinases; DKK, Dickkopf; LLOQ, lower limit of quantification; M-CSF, macrophage colony-stimulating factor.

^a^Data for the histologic evaluation of the placenta were available in 33 (53%) of the 62 women because in 15 cases, the delivery took place at another institution and in 14 cases, histologic evaluation of the placenta was not performed because of our institutional policy that only the placentas in cases of preterm delivery are to be sent for histopathologic examination.

**Fig 1:**
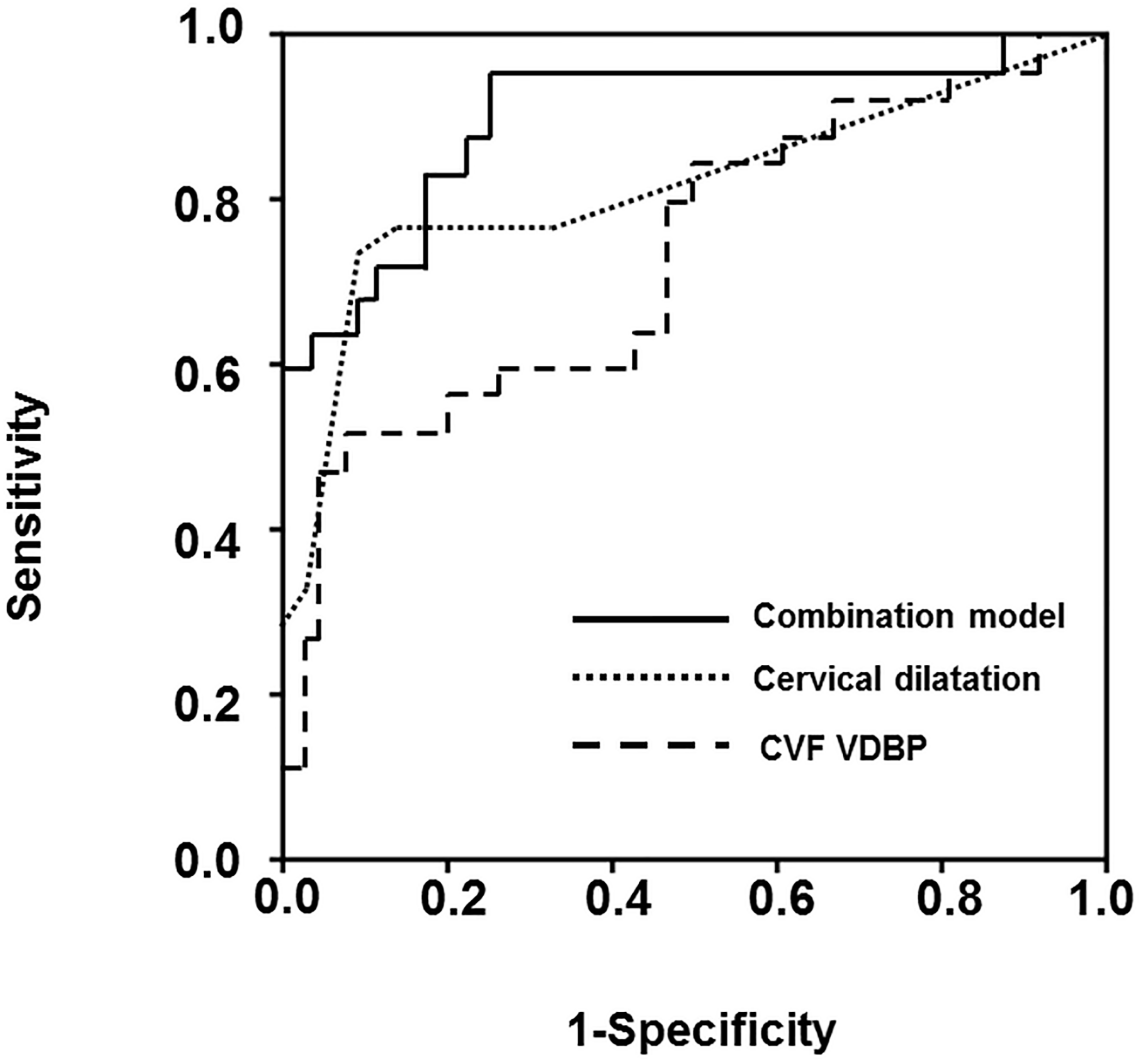
Receiver operating characteristic curves of the CVF VDBP, cervical dilatation, and combined prediction model (including cervical dilatation, CVF VDBP, and use of corticosteroids). The area under the curve for CVF VDBP, cervical dilatation, and combined prediction model was 0.735, 0.818, and 0.909, respectively (*P* = 0.044 between cervical dilatation and the combined prediction model; *P* = 0.005 between CVF VDBP and the combined prediction model). CVF, cervicovaginal fluid; VDBP, vitamin D binding protein.

To develop the best prediction model for SPTD at <32 weeks, proteins levels in the CVF with clinical parameters at enrollment were included in the multivariate analysis. The variables entered into the multivariate analysis were selected based on a *P*-value of <0.05 in the univariate analyses, and the continuous variables were entered into the model as continuous variables because no violation of the linearity assumption was found. The following variables were entered into the forward selection logistic regression model as predictors associated with SPTD at <32 weeks: VDBP, TIMP-1, and DKK3 in the CVF, serum CRP, cervical dilatation, vaginal progesterone therapy, and use of corticosteroids. The final variables retained in the combined prediction model were cervical dilatation, VDBP, and use of corticosteroids {AUC, 0.909 [95% confidence interval (CI) 0.829–0.989]} (Table 2). The Hosmer-Lemeshow test for this model showed a *P*-value of 0.306, indicating an adequate model fit. The use of the cutoff ≥0.23 predicted SPTD at <32 weeks with a sensitivity of 96.0% (95% CI, 79.6–99.9%) and a specificity of 75.7% (95% CI, 58.8–88/2%). The positive and negative likelihood ratios (LRs) were 3.95 (95% CI, 3.2–4.8) and 0.05 (95% CI, 0.007–0.4), respectively. The AUC for the combined predictive model was significantly greater than that for cervical dilatation (*P* = 0.044) and VDBP (*P* = 0.005) (Fig 1).

**Table 2:** Regression coefficients, ORs, and 95% CIs of the final combined model* for predicting SPTD at <32 weeks of gestation.

Predictor	Beta-coefficient	SE	OR (95% CI)	*P*-value
Cervical dilatation (cm)	0.943	0.262	2.56 (1.53–4.29)	<0.001
Cervicovaginal fluid VDBP (μg/mL)	0.875	0.382	2.39 (1.13–5.07)	0.022
Use of corticosteroid	1.866	0.817	6.46 (1.30–32.03)	0.022
Constant	-3.552	0.937	0.029	<0.001

OR, odds ratio; CI, confidence interval; SPTD, spontaneous preterm delivery; SE, standard error; VDBP, vitamin D binding protein; TIMP, tissue inhibitor of metalloproteinases; DKK, Dickkopf; CVF, cervicovaginal fluid; CRP, C-reactive protein. The formula that was generated to predict SPTD at <32 weeks was as follows: Y = logₑ (Z) = -3.552 + 0.943 × [cervical dilatation (in cm)] + 0.875 × [cervicovaginal fluid VDBP (in μg/mL)] + 1.866 × (1 for use of corticosteroid, 0 for non-use of corticosteroid). Z = e^Y^ and risk (%) = [(Z / (1+Z)] × 100.

*Final model resulting from a forward regression analysis including the following predictive parameters: VDBP, TIMP-1, and DKK3 in the CVF, serum CRP, cervical dilatation, vaginal progesterone therapy, and use of corticosteroids.

**Fig 2:**
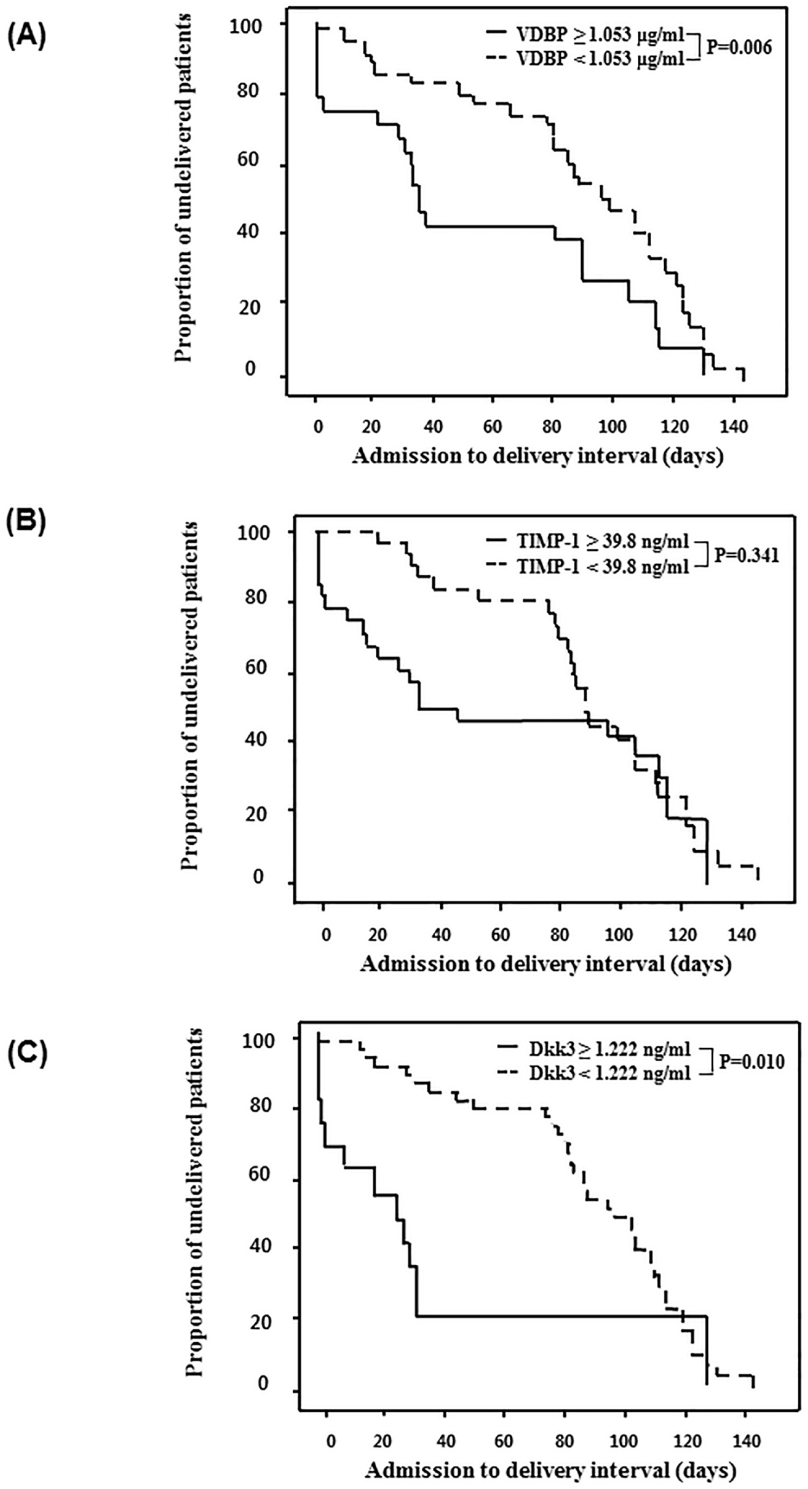
Kaplan-Meier estimates of the sampling-to-delivery interval. For (A) CVF VDBP of ≥1.053 or <1.053 μg/mL, (B) CVF TIMP-1 of ≥39.8 or <39.8 ng/mL, and (C) CVF DKK3 of ≥1.22 or <1.22 ng/mL (CVF VDBP: median, 99.00 days [95% CI, 78.90–119.10] vs. 33.00 days [95% CI, 24.74–41.27]; *P* = 0.006; CVF TIMP-1: median, 88.00 days [95% CI, 81.37–94.63] vs. 46.00 days [95% CI, 0.00–124.97]; *P* = 0.341; CVF DKK3: median, 99.00 days [95% CI, 76.56–121.44] vs. 26.00 days [95% CI, 9.52–42.48]; *P* = 0.010). CVF, cervicovaginal fluid; VDBP, vitamin D binding protein; TIMP, tissue inhibitor of metalloproteinases; DKK3, Dickkopf-related protein 3; CI, confidence interval.

Fig 2 displays the Kaplan-Meier estimates of the sampling-to-delivery interval for the VDBP of ≥1.053 or <1.053 μg/mL, TIMP-1 of ≥39.8 or <39.8 ng/mL, and DKK3 of ≥1.22 or <1.22 ng/mL. Comparisons using the log-rank tests were significant for only VDBP and DKK3 (VDBP ≥1.053, *P* = 0.006; DKK3 ≥1.22 ng/mL, *P* = 0.010).

Table 3 shows the clinical and laboratory characteristics of the study population according to disease entity. The women with cervical insufficiency delivered significantly earlier and had higher risks of preterm delivery before 32 weeks than those with a short cervix. The median CVF levels of IL-6, VDBP, MMP-9, TIMP-1, and DKK3, but not IL-8, were significantly higher in the women with cervical insufficiency than in those with a short cervix.

**Table 3:** Demographic, clinical, and laboratory characteristics of the study population according to disease entity.

	Disease entity		*P*-value
	Cervical insufficiency (n = 27)	Short cervix (n = 35)	
Maternal age (years)	30.9 ± 4.8	33.2 ± 3.5	0.055
Nulliparity	63.0% (17)	38.5% (15)	0.100
Gestational age at sampling (weeks)	22.5 ± 2.6	23.0 ± 2.4	0.996
Gestational age at delivery (weeks)	27.8 ± 6.8	35.4 ± 4.6	<0.001
SPTD at <32 weeks	70.4% (19)	17.1% (6)	<0.001
Cerclage placement	48.1% (13)	60.0% (21)	0.352
Vaginal progesterone therapy	33.3% (9)	54.3% (19)	0.100
WBC count (thousand/mm³)	11.3 ± 3.8	11.0 ± 2.9	0.885
Serum CRP (mg/dL)	1.1 ± 1.5	0.6 ± 1.0	0.176
Cervicovaginal IL-8 (ng/mL)	4.861 ± 2.348	13.885 ± 23.211	0.665
Cervicovaginal VDBP (μg/mL)	1.608 ± 1.502	0.865 ± 0.978	0.030
Cervicovaginal TIMP-1 (ng/mL)	132.78 ± 186.046	46.186 ± 51.369	0.007
Cervicovaginal MMP-9 (ng/mL)	41.618 ± 31.550	74.325 ± 62.627	0.033
Cervicovaginal DKK3 (ng/mL)	2.384 ± 2.384	0.537 ± 1.362	<0.001
Percentage above LLOQ for cervicovaginal IL-6	77.8% (21)	25.7% (9)	<0.001
Percentage above LLOQ for cervicovaginal M-CSF	48.1% (13)	40.0% (14)	0.521
Use of antibiotics	85.2% (23)	77.1% (27)	0.427
Use of tocolytics	44.4% (12)	40.0% (14)	0.725
Use of antenatal corticosteroids	48.1% (13)	34.3% (12)	0.270
Clinical chorioamnionitis	18.5% (5)	2.9% (1)	0.077
Histologic chorioamnionitis^a^	75.0% (15/20)	38.5% (5/13)	0.036

Values are given as means ± SDs or % (n). SPTD, spontaneous preterm delivery; WBC, white blood cell; CRP, C-reactive protein; IL, interleukin; VDBP, vitamin D binding protein; TIMP, tissue inhibitor of metalloproteinases; MMP, matrix metalloproteinases; DKK, Dickkopf; LLOQ, lower limit of quantification; M-CSF, macrophage colony-stimulating factor.

^a^Data for the histologic evaluation of the placenta were available in 33 (53%) of the 62 women because in 15 cases, the delivery took place at another institution and in 14 cases, histologic evaluation of the placenta was not performed because of our institutional policy that only the placentas in cases of preterm delivery are to be sent for histopathologic examination.

When separate analyses were performed on the dataset after removing 5 women who developed clinical chorioamnionitis before the onset of labor, the results were almost the same as in the present analyses (data not shown).

## Discussion

The principal findings of this study are as follows: (i) In women with cervical insufficiency or a short cervix, VDBP, TIMP-1, and DKK3 in the CVF may be useful as non-invasive predictors of SPTD at <32 weeks; (ii) a combination of these markers and clinical factors appears to improve predictability of SPTD in comparison to the markers alone; and (iii) the median CVF levels of VDBP, MMP-9, TIMP-1, and DKK3 were significantly higher in the women with cervical insufficiency than in those with a short cervix. To our knowledge, this is the first study to demonstrate the distinctive changes in various proteins present in the cervicovaginal compartment between women with cervical insufficiency/short cervix who had SPTD at <32 weeks and delivered 32 weeks later. Similar results regarding pro-inflammatory cytokines and VDBP were also documented in the CVF of women with preterm labor and intact membranes [6, 8, 13].

The present study has several limitations. The major limitation of the study is that the study population constituted a heterogeneous group in terms of disease entities (i.e., cervical insufficiency and short cervix) and treatment (i.e., the use of progesterone therapy and placement of a cervical cerclage), despite the fact that a short cervix cannot be equated with cervical insufficiency. However, it is unlikely that this has changed our main findings because we adjusted for these confounders in the multivariate analysis. Second, this study is limited by its retrospective nature and relatively small sample size from a single center. Data from the current study may provide pilot information with regard to concentrations and role of various proteins present in the CVF compartment in cases of cervical insufficiency. Therefore, our finding should be confirmed by prospective studies with larger population sizes. Third, we included women who presented with cervical insufficiency at an advanced gestational age (beyond 26 weeks, n = 3) to increase the sampler size, even though the assessment of cervical insufficiency beyond 26 +0 weeks is too late to be clinically relevant. Fourth, this study lacked data on the CVF fetal fibronectin test, which has been reported to be useful in predicting SPTD in asymptomatic women and women with preterm labor [25, 26]. Therefore, the present study did not compare the effectiveness of the proteins measured in the CVF with that of the CVF fetal fibronectin test for the prediction of SPTD.

The present study demonstrates that a combination of various proteins in the CVF and clinical factors can improve the predictability of SPTD, and this combination is more accurate than these factors alone. These observations are consistent with the results of our previous studies on multiple proteins in the amniotic fluid (AF) and serum compartments in cases of cervical insufficiency [20, 27]. Collectively, these observations suggested that the etiology of SPTD occurring in cervical insufficiency may be multifactorial. With respect to the combination of the best prediction of SPTD, a combination of CVF VDBP, cervical dilatation at presentation, and use of corticosteroid is the best combination to predict SPTD at <32 weeks of gestation, showing a sensitivity of 96.0% (24/25), specificity of 75.7% (28/37), positive predictive value of 72.7% (24/33), and negative predictive value of 96.6% (28/29), using a cutoff value of 0.23. Considering its high sensitivity and negative predictive value, this combination is a more valuable test when the results are negative (justly rules out SPTD), suggesting an effective identification of women with cervical insufficiency at the lowest risk for SPTD. Notably, we found that the use of corticosteroid included in the prediction model was significantly associated with an increased risk of SPTD at <32 weeks. This finding is unexpected; thus, it may only be a chance finding because the current study has a small sample size. However, considering the reports that the administration of glucocorticoids initiated parturition in several domestic mammalian animal species [28], in prolonged human pregnancy [29], and in high-order multiple gestations in humans [30], it may be postulated that the impact of glucocorticoids used may be a variable of the risk of SPTD according to the types of obstetric complications. Further large prospective studies, especially in asymptomatic women at high risk of preterm birth, are needed to confirm our findings.

Consistent with the findings of previous studies on women with preterm labor [12, 13], an elevated VDBP level in the CVF appeared to be predictive of SPTD in our population. These findings are not surprising given that 1) an increased synthesis of VDBP can be stimulated by the presence of IL-6, *in vitro* [31], of which elevated levels in the AF and CVF have been reported to be the strongest predictor of SPTD [6, 8, 27] and that 2) the VDBP is a protein in which specific binding sites are expressed on the surface of inflammatory cells and the fibroblast plays an important role in the extracellular remodeling of the cervix [32, 33]. In fact, it has been shown in the literature that the VDBP is a multi-functional plasma protein that can play a certain role in the modulation of immunity and inflammatory responses, although it acts as a carrier protein for vitamin D and its metabolites [32, 34]. Meanwhile, we found that there was a significant association of CVF TIMP-1 with SPTD; however, CVF MMP-9 did not significantly contribute to SPTD. Similarly, in cases of cervicovaginal secretions of women at term, Heng et al. also have reported similar results to those in our study in the prediction of term labor [9]. In contrast to the CVF samples, previous studies on AF samples have reported that MMP-9, but not TIMP-1, has been implicated in association with spontaneous labor both at term and preterm [7, 10]. We cannot explain the discrepancy in MMP-9 and TIMP-1 production observed between the CVF and AF compartments. Nonetheless, our observations and those of others suggest that an increased availability of TIMP-1 in the CVF and MMP-9 in the AF is part of the common terminal pathway of preterm and term parturition [7, 9, 10]. Our finding that SPTD was not associated with an increased CVF IL-8 is in contrast with the results of Sakai et al., who demonstrated a significant association between premature delivery and high IL-8 levels in the cervical mucus among patients with cervical shortening [35]. The discrepancy between these two studies may be because of the differences in the swabbing site (cervical mucus versus CVF), sample size (246 versus 62), and population studied (women with short cervix versus those with cervical insufficiency/short cervix).

In our study, CVF DKK3 displayed the strongest association with SPTD among the measured biomarkers (see the supplementary files). Although DKK3 is a relatively less-studied protein particularly in obstetrics, the highest DKK3 levels are found in organs classically considered as immune privileged, such as the embryo, placenta, and uterus [15, 36]. Importantly, recent reports demonstrated that DKK3 functions as a tissue-derived modulator of T-cell responses as well as a modulator of B-cell fate and maintenance [37, 38], suggesting that DKK3 may play an important role in the final common pathway of term and preterm parturition [39]. Therefore, DKK3 can be a potentially valuable target for diagnostic, prognostic, and therapeutic approaches to threatened preterm birth related to infectious and noninfectious etiologies; thus, this will be confirmed in the CVF, AF, and serum samples by further studies.

We also found that the levels of VDBP, MMP-9, TIMP-1, and DKK3 and percentage above the limit of detection for IL-6 in the CVF were significantly higher in the women with cervical insufficiency than in those with a short cervix. In addition, we have found a significant correlation between cervical dilatation and these protein levels in the CVF, except for MMP-9. Similarly, in the context of the AF compartment, previous reports by Kiefer et al. and our group have shown a significant correlation between inflammatory cytokine levels in the AF and the degree of cervical shortening in women presenting with mid-trimester short cervix or the degree of cervical dilatation in women with cervical insufficiency [20, 40]. When taken together, these findings suggest that inflammatory and immune regulatory proteins in both the CVF and AF compartments may play an important role in the progression of cervical insufficiency/short cervix occurring along a continuum of severity. In cervical insufficiency considered as a clinical entity having multiple etiologies, these novel biomarkers in the current study can help elucidate the underlying pathophysiologic mechanism of its development, leading to better treatment that can ultimately improve patient outcomes.

## Conclusions

In conclusion, VDBP, TIMP-1, and DKK3 in the CVF may be useful as non-invasive predictors of SPTD in women with cervical insufficiency or a short cervix. A combination of these markers and clinical factors appears to improve the predictability of SPTD compared with markers alone. Further large prospective studies are needed to determine whether our prediction model as a management strategy can be used to identify the patients who would benefit from cerclage.

## Supporting information

S1 Table Areas under the ROC curves and best cutoff values for every protein in the cervicovaginal fluid in relation to the occurrence of spontaneous preterm delivery at <32 weeks. The AUC values with their 95% CIs and best cutoff values of VDBP, TIMP-1, and DKK3 in the CVF and serum CRP in relation to the occurrence of SPTD at <32 weeks. The AUCs for these three proteins in the CVF ranged from 0.735 to 0.799, which were not significantly different from each other (all variables: *P* = 0.300–0.879). ROC, receiver operating characteristics; SE, standard error; CI, confidence interval; VDBP, vitamin D binding protein; TIMP, tissue inhibitor of metalloproteinases; DKK, Dickkopf ^a^Comparison with cervical dilatation.
(DOCX)

S1 File Raw data. (SAV)
