# Supplementary material for: Non-invasive prediction of preterm birth in women with cervical insufficiency or an asymptomatic short cervix (≤25 mm) by measurement of biomarkers in the cervicovaginal fluid
Source: PLoS One. 2017 Jul 10;12(7):e0180878. doi: 10.1371/journal.pone.0180878 (PMC5507270; doi:10.1371/journal.pone.0180878)
Supplement: S1 Table — The AUC values with their 95% CIs and best cutoff values of VDBP, TIMP-1, and DKK3 in the CVF and serum CRP in relation to the occurrence of SPTD at <32 weeks. The AUCs for these three proteins in the CVF ranged from 0.735 to 0.799, which were not significantly different from each other (all variables: P = 0.300–0.879). ROC, receiver operating characteristics; SE, standard error; CI, confidence interval; VDBP, vitamin D binding protein; TIMP, tissue inhibitor of metalloproteinases; DKK, Dickkopf aComparison with cervical dilatation. (DOCX) [file pone.0180878.s001.docx]

**S1 Table.** Areas under the ROC curves and best cutoff values for every protein in the cervicovaginal fluid in relation to the occurrence of spontaneous preterm delivery at <32 weeks

|  | Area (± SE) under the ROC curve | 95% CI | Cutoff  value | *P*-value^a^ |
| --- | --- | --- | --- | --- |
| Cervical dilatation (cm) | 0.818 ± 0.061 | 0.698–0.938 | 2 | Reference |
| Cervicovaginal VDBP (μg/mL) | 0.735 ± 0.066 | 0.605–0.864 | 1.053 | 0.353 |
| Cervicovaginal TIMP-1 (ng/mL) | 0.744 ± 0.064 | 0.619–0.869 | 39.8 | 0.376 |
| Cervicovaginal DKK3 (ng/mL) | 0.799 ± 0.060 | 0.682–0.917 | 1.222 | 0.728 |
| Serum C-reactive protein (mg/dL) | 0.732 ± 0.065 | 0.605–0.859 | 0.365 | 0.339 |

ROC, receiver operating characteristics; SE, standard error; CI, confidence interval; VDBP, vitamin D binding protein; TIMP, tissue inhibitor of
metalloproteinases; DKK, Dickkopf

^a^Comparison with cervical dilatation
